# Feasibility of a Small Group Otago Exercise Program for Older Adults Living with Dementia

**DOI:** 10.3390/geriatrics7020023

**Published:** 2022-02-24

**Authors:** Julie D. Ries, Martha Carroll

**Affiliations:** Center for Optimal Aging, Physical Therapy Department, School of Health Sciences, College of Health & Education, Marymount University, Arlington, VA 22207, USA; mcarroll@marymount.edu

**Keywords:** dementia, balance, falls, exercise, Otago Exercise Program

## Abstract

Older adults with dementia experience more frequent and injurious falls than their cognitively-intact peers; however, there are no evidence-based fall-prevention programs (EBFPP) for this population. The Otago Exercise Program (OEP) is an EBFPP for older adults that has not been well-studied in people with dementia. We sought to explore the feasibility of group delivery of OEP in an adult day health center (ADHC) for people with dementia. We collected demographic data, Functional Assessment Staging Tool (FAST), and Mini Mental State Exam (MMSE) scores for seven participants with dementia. Pre- and post-test data included: Timed-Up-and-Go (TUG), 30-Second Chair-Stand (30s-CST), Four-Stage-Balance-Test (4-SBT), and Berg Balance Scale (BBS). We implemented a supervised group OEP, 3x/week × 8 weeks. Most participants required 1:1 supervision for optimal challenge and participation. Five participants completed the program. All had moderately severe to severe dementia based upon FAST; MMSE scores ranged from mild to severe cognitive impairment. Four of five participants crossed the threshold from higher to lower fall risk in at least one outcome (TUG, 30s-CST, 4-SBT, or BBS), and four of five participants improved by >Minimal Detectible Change (MDC_90_) score in at least one outcome. The group delivery format of OEP required significant staff oversight for optimal participation, making the program unsustainable.

## 1. Introduction

Exercise alone, or as a component of a comprehensive fall-prevention program, can effectively decrease falls in older adults [1,2,3,4]. There is cautious optimism for exercise interventions to reduce falls in individuals with dementia (IwD) [5], but there are no identified evidence-based fall-prevention programs (EBFPPs) for this population [4,6,7]. IwD display an increased prevalence and frequency of falls, and are more likely to be seriously injured from a fall than their cognitively-intact age-matched peers [8,9,10]. The Otago Exercise Program (OEP) [11], comprised of strength and balance exercises and a walking program, is one of the EBFPPs supported by the STEADI (Stopping Elderly Accidents, Deaths, and Injuries) initiative of the US Center for Disease Control and Prevention (CDC) [12]. The OEP has not been well-studied with IwD. Suttanon et al. [13] found an in-home OEP-based program to be feasible and safe for participants with mild to moderate Alzheimer disease (MMSE mean score 21/30). A retrospective study by Trappuzano et al. [14] showed some benefit of home health administered OEP for people with dementia (miniCog mean score of 1.76/5), and Beato et al. [15] and Knott et al. [16] retrospectively found a positive impact of an OEP-based intervention for assisted-living residents without commenting on the cognitive status of participants.

Group administration of OEP is beginning to be represented in the literature. Kocic et al. [17] provided OEP in a nursing home setting, where two physiotherapists oversaw groups of up to ten participants, but they intentionally excluded those with cognitive impairment. Renfro et al. [18] demonstrated the feasibility of a community-based group OEP with adults with intellectual disabilities. In long-term care, Kovàcs et al. [19] compared group OEP to no exercise for older adults with cognitive impairment (mean MMSE 21/30) and demonstrated improvements in balance, but not falls, in the OEP group. Any enthusiasm for “group” OEP is quelled with careful reading of these studies: Renfro et al. averaged classes of 8 participants with the help of “2–3 caregivers/staff, 5–7 nursing students, and 1 or 2 PI/PTs” [18] (p. 4) (it appears there may have been days where the help outnumbered the participants!), and Kovàcs et al. delivered the program with two physiotherapists for groups of 2–4 participants [19]. Recently, a series of studies in nursing home settings has supported the feasibility of group OEP with residents with physical and cognitive frailty [20,21,22,23]. While the mean MMSE scores of participants represent cognitive impairment (21–22/30) [20,21], a diagnosis of dementia was not in the inclusion criteria of any of these studies. The strategy for group administration of OEP in two of these studies was to initiate the program in small groups (6 participants to 3 staff [21] or 4–7 participants to 1 staff [22]) and after 4 weeks, to combine all small groups into one larger group with 3 staff providing oversight to groups of 18 and 29, respectively. The mechanics of group OEP administration in the other studies (i.e., size of group and number of staff) was not presented [20,23].

The purpose of this communication is to share findings of a small feasibility study for group OEP for IwD at an Adult Day Health Center (ADHC) and, secondarily, to provide insights on group exercise for IwD. Most ADHC settings provide seated group exercise activities, but not standing/upright exercises, as staff must cater to the lowest level of functioning for safety and maximal participation. There is benefit to physical therapist (PT)-directed exercise programs in improving balance in participants of ADHC [24,25], but we are striving for a program that can be sustained with consultation from, rather than direct oversight by, a PT.

OEP [11,26] starts with a series of five warm-up/flexibility exercises for the head/neck, trunk, and ankles. Strength-training exercises include seated knee extension, ankle dorsiflexion and plantar flexion, and standing knee flexion and hip abduction. Cuff weights provide resistance and are increased when the participant completes 2 sets of 10 repetitions without complaint. Balance exercises are performed in an upright position (standing or walking) and are progressed by decreasing upper extremity (UE) support. They include partial squats, sit to stand, sideways walking, backward walking, walking with turns, single limb stance, tandem stance, heel-toe walking, walking on heels, walking on toes, backward heel–toe walking, and stair climbing (we omitted stair climbing as we did not have easy access to stairs).

OEP is imperfect as a comprehensive balance training program as it is solely driven by anticipatory balance challenges of base-of-support/center-of-gravity manipulation. We were willing to forego balance training with a more robust design (i.e., inclusive of changing visual (eyes closed, scanning environment) and somatosensory experience (walking and balancing on foam and other altered surfaces), reactive challenges (perturbations, prop use), deliberate dual tasking (superimposing cognitive and physical tasks)), in exchange for a program transferrable to ADHC oversight. The prescriptive nature of OEP, replete with manual and pictures, was considered an asset of the program.

## 2. Materials and Methods

This exploratory study is categorized as an “intervention development” feasibility study [27], a preliminary step to determine if a formal feasibility study of group OEP that can be sustained by ADHC staff is realistic and appropriate. The study was approved by the Marymount University Institutional Review Board (MUIRB#387) and written informed consent was provided by legal proxy decision-makers.

A sample of convenience of seven individuals at a local county-sponsored ADHC met the inclusion criteria of: physician diagnosis of dementia, medical stability, ability to walk without physical assistance (assistive device acceptable), and ability to follow one-step commands. Exclusion criteria included: unstable or limiting systemic pathology; any surgery or cancer diagnosis or treatment within previous six months. Demographic data, collected from facility health records, are presented in Table 1. Mini-Mental Status Examination (MMSE) [28] and the Functional Assessment Staging Tool (FAST) [29] were administered by the facility nurse. A score of ≤23 on the MMSE is the generally accepted indicator of cognitive impairment, with 18 to 23 indicating mild impairment and 0 to 17 indicating more severe impairment [30]. The FAST reveals functional implications of dementia on daily life.

Measures of balance and gait were assessed within two weeks prior to, and one week following, the intervention. Informed by the STEADI initiative [31], we administered: Timed Up-and-Go (TUG), 30 s Chair Stand Test (30s-CST), and 4-Stage Balance Test (4-SBT). Additionally, participants performed the Berg Balance Scale (BBS). Tests were performed in the order presented below and were scored onsite by the tester and scored on video by a second PT. On the rare occasion that scores were not corroborated, the therapists came to consensus on the final score.

For the TUG [32], participants began seated in a chair with arm rests, were instructed to walk “quickly, but safely”, and on the instruction “Go”, stood, walked 3 m, circled around a cone, walked back to the chair, and sat down. As has been previously documented [33], timing began with movement initiation as opposed to the “Go” command and finished when the buttocks contacted the chair. Cuing was provided as needed (e.g., “go around the cone”, “sit in that chair”). The score was the mean time of two trials. The OEP Manual [34] suggests that the cutoff score of ≥12 s indicates a higher risk of falls, consistent with evidence by Lusardi et al. [35].

The 30s-CST [36] is the number of stands completed in 30 s without the use of arms. A 17-inch seat-to-floor height chair was used, and cuing was offered throughout the test as needed, encouraging continued efforts [37]. The OEP Manual [34] suggests individuals performing below age and sex-matched norms are at higher risk of falls, consistent with evidence that LE strength is associated with fall risk [4].

The 4-SBT requires balancing in each of 4 foot positions for 10 s (feet together, semi-tandem, tandem, single-limb stance) and is based on the FICSIT-4 (Frailty and Injuries: Cooperative Studies of Intervention Techniques) [38], which uses the same foot positions with more complex scoring. There is some precedent for use of the 4-SBT [14] and FICSIT [39,40] in dementia research. Evidence that community living older adults unable to maintain tandem stance ≥10 s are at higher risk of falls [41] makes the inability to sustain position 3 (tandem) an indication of fall risk in the OEP Manual [34].

The BBS consists of 14 progressively challenging, functionally oriented balance activities, ranging in difficulty from sitting unsupported to turning in a circle and single-limb stance. Each item is scored from 0 (unable to perform) to 4 (proficient performance). The BBS has been used with older adults with cognitive impairment [42,43]. Lusardi et al. [35] identify <50 as a cutoff score for fall risk in community-dwelling older adults.

The exercise intervention was an 8 week, 3x/week, 45 min class following the OEP curriculum [11,26]. The PTs overseeing exercise classes completed online OEP instructor training [26] and were well-versed in OEP administration and program fidelity; student instructors were trained by these PTs. All instructors were trained in communication and rehabilitation principles to facilitate optimal success with IwD [44]. The program was performed in the therapy room of the ADHC at a consistent time with a consistent PT instructor, occasional support of a second PT, and student instructors (nursing and PT students). Music, chosen by participants, was played during the class. The goal was to have one instructor oversee as many participants as possible, providing it was safe and effective. Based on our experience in this venue, we planned to oversee a class of 9 participants with 3 instructors; with 5 participants, we envisioned 2 instructors would suffice. It quickly became apparent that optimal engagement (i.e., participants consistently performing exercises at a level of appropriate challenge) would require closer oversight. Initially, when we attempted to run classes with fewer instructors, the time/repetition per task and intensity of challenge all suffered. It was never unsafe, just ineffective. Without a “personal instructor”, attention waned and the tendency for self-challenge was feeble in all but one participant (Participant 1). Ultimately, we used at most 1:2, but usually 1:1 supervision. The staff of the ADHC oversaw the walking component of the program and charted participation.

## 3. Results

While participants sometimes exercised during our class without 1:1 supervision, they rarely challenged themselves or sustained the activity independently. When participants were not maximally engaged, student instructors stepped in to assist, which inevitably enhanced participation. We made efforts to wane supervision repeatedly throughout the 8 week intervention, but this resulted in reversion to less effort and/or attention from participants. Prioritizing safety, we had 1:1 supervision with our most physically impaired participants who were at highest risk for falls (Participants 2 and 4). Participants 3 and 5 required constant and focused supervision to remain cognitively engaged. Participant 1 was the only participant who did not require constant 1:1 supervision for optimal participation; thus, he was usually paired with Participant 2 (staff could provide physical assistance to Participant 2 and verbal cuing to Participant 1).

Seven participants underwent pre-testing and two withdrew prior to the intervention due to unrelated medical issues. The withdrawn participants were slightly younger and slightly higher in MMSE scores, as evidenced in comparing means/median scores of all participants (*n* = 7) vs. only those who completed the intervention (*n* = 5) (Table 1). All participants, save one, rated FAST Stage 6 (“moderately severe dementia”), demonstrating difficulty with the pragmatics of dressing, bathing, and toileting. Participant 3 was rated Stage 7 (“severe dementia”), as evidenced by a dependency on ADLs and minimally preserved speech. Participant data were analyzed by comparing changes in pre- and post-test performance with established Minimal Detectible Change scores at the 90% confidence interval (MDC_90_) for older adults with dementia. A change score exceeding the MDC_90_ score is thought to represent a “true” change (i.e., beyond what would be expected from individual variability and/or measurement error). The MDC_90_ score used for the TUG was 4.09 s [33]; for the 30s-CST, it was 2 repetitions [37]; and for the BBS, it was 6.4 [45]. While the 4-SBT has been used in dementia research, the published MDC_95_ score for this population of 1.5 units was based on the FICSIT scoring model, and therefore does not translate to 4-SBT [40]. In determining outcomes related to 4-SBT, we focused on position 3 (tandem stance) as a cutoff score and looked for changes in performance from <3 to ≥3 to represent a clinically meaningful change.

Outcomes are presented in Table 2. One participant (Participant 3) improved TUG performance by >MDC_90_, and four participants improved their 30s-CST by ≥MDC_90_. Participants 3 and 4 not only improved by MDC_90_ but hit the threshold for age/sex matched norms for 30s-CST, indicating a transition from higher to lower fall risk. Participant 1 performed well above the norm for 30s-CST, and Participants 3 and 5 showed improvement within the norm spectrum. Three of the five participants who failed tandem stance in the 4-SBT pre-test passed the post-test, which in the context of fall risk is considered an important milestone [41]. There were no adverse events during testing or interventions. There were two reported non-injurious falls at home over the course of the study, both by Participant 2. Participant 4’s spouse became an unofficial participant and official volunteer with the program over the course of the 8 week intervention.

## 4. Discussion

The purpose of this preliminary study was to determine the feasibility of a group OEP for IwD that could be sustained by ADHC staff. In facilitating progress from a physiologic and motor learning standpoint, the importance of maximizing engagement and challenge for individual participants is an important consideration [46]. The need for four staff members to provide an optimal challenge and engagement of five participants was not sustainable. Maximizing the challenge did not appear to be a priority in recent published studies, which either do not comment on efforts to individualize the intervention [21,22] or allude to progressing the group as a whole [23]. Chen et al. [20] mention that physiotherapists “guided” participants to modify their exercise level, but the weights used were consistent at 0.5 kg and nurses “led” the intervention, so it is not clear how often physiotherapists may have guided these changes.

In comparison to other studies of group OEP administration [19,20,21,22], our participants were more cognitively impaired (diagnosis of dementia, mean MMSE of 16), and notably, the most physically impaired (forcing 1:1 supervision for safety) were the most cognitively capable. This contributed to our increased staff requirements. Existing studies, without commenting on efforts to maximize engagement and challenge of participants, identify beneficial outcomes of group OEP [20,21,22,23]. In reconsidering the feasibility of group administration of OEP, clear prioritization of safety over maximizing challenge may decrease the need for staff supervision.

One might assume that the need for such close supervision for optimal engagement negates the benefit of an exercise class (i.e., why not just work independently with participants?), but we perceived a strong social benefit from interactions within each class. A recent systematic review suggests that exercise administered in group format for IwD is associated with higher adherence rates [47]. An atmosphere of joy was a deliberate component of our intervention, and smiling, laughing, and joking were routine occurrences. Participants’ music preferences drove the class playlist each day, creating the opportunity to hear and talk about personal favorites ranging from Latin instrumental to the Rolling Stones! Gonҫalves et al. [48], in striving to establish core outcome measures, determined that the outcome most frequently sought by individuals with dementia and their caregivers was “enjoyment”. Fun has been identified as a relevant characteristic of group exercise classes for fall prevention in older adults and, in fact, immediate enjoyment and social interaction may be more influential in motivating older adults to exercise than the promise of decreasing the fall risk [49]. Booth et al. [50], in a review of exercise-based fall-prevention programs, highlight the relevance of enjoyment in the context of motivating IwD, who may not be as self-actualized as their cognitively-intact peers. The benefit of a shared exercise experience is consistent with the findings of Lindelöf et al. [51], who, in a qualitative study of participants with dementia, found a theme of “Togetherness gives comfort, joy, and encouragement”. A second theme in their study, “Exercise evokes body memories”, was also represented in our study, as participants spoke of their younger life physical capabilities with pride and fondness. One participant reminisced about being a competitive track athlete, and another spoke of her days as a physical education teacher and coach. They were encouraged to tap into these memories throughout the class.

Over the course of the study, four of five participants crossed the threshold from higher to lower fall risk in at least one outcome, and four of five participants improved by >MDC_90_ change score in at least one outcome. Given the substantial methodological limitations of the small sample size and lack of control group, we cannot draw any conclusions about the impact of OEP. We did learn that our framework for bringing group OEP to ADHC is not feasible, as it is not sustainable. Our intent was to have the ADHC sustain the program, such that the dosage would reach recommended levels for older adults of >50 h over 6 months (i.e., 2 h/week), providing the best opportunity for fall reduction impact [52]. Instead, at the completion of this study, the OEP class participants rejoined the seated exercise group activity.

One consideration in sustaining balance training for this population is the benefit of educating and engaging caregivers, and working as a team (clinician, family/caregiver, patient/participant) to facilitate ongoing efforts. Meyer et al. [53] demonstrate that effective knowledge translation of fall-prevention strategies requires an inclusive approach that respects and understands the perspective of the person with dementia and their caregiver. The wife of Participant 4 was a regular ADHC volunteer and became an unofficial member of the class on her volunteer days. She was motivated by the experience to continue OEP with her husband at home when the study concluded and felt that she and her husband were both benefitting. Engaging dyads of IwD and care partners in OEP classes may be a future research direction.

## 5. Conclusions

The administration of a small group OEP 3x/week for 8 weeks with the prioritization of maximizing individual engagement and challenge was not feasible for ADHC sustainability. The perceived social benefit of group delivery encourages us to continue searching for sustainable group balance training options for ADHC participants.

## Figures and Tables

**Table 1:** Participant characteristics.

	Participants								
	1	2	3	4	5	6	7	Mean/Median All Participants *n* = 7	Mean/Median Completers *n* = 5
Sex	M	F	F	M	F	F	F	71% Female	60% Female
Age (years)	85	89	90	72	89	79	83	83.9	85
MMSE Score	17/30	20/30	6/30	24/30	14/30	22/30	20/30	Mean = 17.6 Median = 20	Mean = 16.2 Median = 17
FAST Score	6A	6B	7A	6C	6A	6D	6C	Median = 6	Median = 6
Comorbidities	8 (a [3], b [2], f, g [2])	6 (a [2], b, d, h [2])	5 (a [2], d [2], d)	4 (a, c, e, g)	4(a [3], g)	5 (a [2], b, c, e)	7 (a [2], b, c, d, e, f)	5.6	5.4
Medications	5	5	6	7	4	6	6	5.6	5.4
Fall in Past Year	No	Yes	Yes	Yes	No	Yes	No	N/A	N/A
Assistive Device	None	RW	None	None	None	Cane	RW	N/A	N/A
Attendance	96%	96%	65%	74%	96%	0	0	N/A	85.4%

MMSE = Mini Mental Status Exam; FAST = Functional Assessment Staging Tool (A through D represent progressive levels of impairment); RW = rolling walker. Comorbidities: a = cardiovascular disease (e.g., hypertension, hypercholesterolemia, atrial fibrillation, orthostatic hypotension, syncope); b = endocrine/thyroid disorder (e.g., diabetes, hypothyroid); c = neuro disorder (e.g., Parkinson’s disease, stroke, seizure disorder); d = ortho disorder (e.g., osteoporosis, arthritis); e = psychological/psychiatric disorder (e.g., anxiety, depression); f = vision disorder (e.g., cataracts, macular degeneration); g = gastrointestinal/genitourinary disorder (e.g., GERD, chronic constipation, kidney disease); h = history of cancer.

**Table 2:** Participant outcomes.

	Participant 1			Participant 2			Participant 3			Participant 4			Participant 5		
	Pre-Test	Post-Test	Δ Score	Pre-Test	Post-Test	Δ Score	Pre-Test	Post-Test	Δ Score	Pre-Test	Post-Test	Δ Score	Pre-Test	Post-Test	Δ Score
TUG (sec)	10.5	11.6	(1.1)	48.5	46.1	2.4	25.0	17.6	7.4 ^a^	11.3	9.4	1.9	13.5	14.8	(1.3)
30s-CST (reps)	16	20	4 ^a^	0	0	0	6	9	3 ^a^	9	15	6 ^ab^	8	10	2 ^a^
4-SBT	2	3	1 ^b^	2	2	0	2	3	1 ^b^	2	2	0	2	3	1 ^b^
BBS	51	54	3	27	24	(3)	44	45	1	41	43	2	52	51	(1)
Super-vision needs	Paired with another participant (2) for oversight Frequent verbal cuing required for optimal level of challenge			Required 1:1 oversight Constant physical assistance required for safety and optimal level of challenge			Required 1:1 oversight Constant physical assistance and verbal cuing required for safety and optimal level of challenge			Required 1:1 oversight Constant physical assistance and verbal cuing required for safety and optimal level of challenge			Required 1:1 oversight Constant physical presence and verbal cuing required for optimal level of challenge		

Δ Score = change score, TUG = Timed Up-And-Go, 30s-CST = 30-s Chair Stand Test, 4-SBT = 4 Stage Balance Test, BBS = Berg Balance Scale. Parenthetical numbers show negative change, none of which met MDC_90_ and therefore represent no “true” change in performance. ^a^ = Performance change meets minimal detectable change (MDC_90_) score; shows true positive change (improved performance in post-test). ^b^ = Performance change represents change from higher to lower fall risk per OEP guidelines (i.e., from fail to pass Position 3 (tandem stance) in 4-SBT, from below to within age & sex matched norms for 30s-CST).

## Data Availability

Data for this pilot study are available from the corresponding author.
